# Community Structure of a Mental Health Internet Support Group: Modularity in User Thread Participation

**DOI:** 10.2196/mental.4961

**Published:** 2016-05-30

**Authors:** Bradley Carron-Arthur, Julia Reynolds, Kylie Bennett, Anthony Bennett, John Alastair Cunningham, Kathleen Margaret Griffiths

**Affiliations:** ^1^ National Institute for Mental Health Research Research School of Population Health The Australian National University Acton Australia; ^2^ Centre for Addiction and Mental Health Toronto, ON Canada

**Keywords:** internet, support group, social network, modularity, mental health, super user

## Abstract

**Background:**

Little is known about the community structure of mental health Internet support groups, quantitatively. A greater understanding of the factors, which lead to user interaction, is needed to explain the design information of these services and future research concerning their utility.

**Objective:**

A study was conducted to determine the characteristics of users associated with the subgroup community structure of an Internet support group for mental health issues.

**Methods:**

A social network analysis of the Internet support group BlueBoard (blueboard.anu.edu.au) was performed to determine the modularity of the community using the Louvain method. Demographic characteristics age, gender, residential location, type of user (consumer, carer, or other), registration date, and posting frequency in subforums (depression, generalized anxiety, social anxiety, panic disorder, bipolar disorder, obsessive compulsive disorder, borderline personality disorder, eating disorders, carers, general (eg, “chit chat”), and suggestions box) of the BlueBoard users were assessed as potential predictors of the resulting subgroup structure.

**Results:**

The analysis of modularity identified five main subgroups in the BlueBoard community. Registration date was found to be the largest contributor to the modularity outcome as observed by multinomial logistic regression. The addition of this variable to the final model containing all other factors improved its classification accuracy by 46.3%, that is, from 37.9% to 84.2%. Further investigation of this variable revealed that the most active and central users registered significantly earlier than the median registration time in each group.

**Conclusions:**

The five subgroups resembled five generations of BlueBoard in distinct eras that transcended discussion about different mental health issues. This finding may be due to the activity of highly engaged and central users who communicate with many other users. Future research should seek to determine the generalizability of this finding and investigate the role that highly active and central users may play in the formation of this phenomenon.

## Introduction

Online peer-support is a popular source of health information and social support. Findings suggest that in a 1-year period, 18% of the Internet users in the USA sought information online that was provided by a peer [1] and 8% actively sought a response or provided support to another peer by engaging in an online health community [2]. Annually, 28% of Internet users have sought mental health specific information online [3]. Consequently, Mental Health Internet Support Groups (MHISGs) can comprise thousands of users who are actively participating to varying degrees [4,5]. MHISGs are popular and have high potential to play a role in the management of mental illness. Research on MHISGs must address various questions concerning the nature of MHISGs such as “Who uses them?” and “How are they used?” in order to fully benefit from this potential [6]. Recent research on the demographic characteristics of MHISG users has identified differences in prevalence, engagement, and retention of users with different characteristics, such as age, gender, location and consumer or carer status [Personal communication by Kathleen M Griffiths, 2016]. This information is important in understanding to whom do MHISGs have greater appeal. The willingness to engage and the outcome of participating in the MHISG may be different for each user depending on whom they interact with, however, it is also important to understand the social dynamics of how users engage with each other.

From a sociological perspective, the principle of homophily suggests that those who group together, in this instance by communicating most often with each other, tend to share common characteristics [7]. If the premise of peer-support is a shared experience, then it is plausible that homophily may be an important underlying factor in the community structure of the MHISG, that is, the community structure of the MHISG may comprise various subgroups, each consisting of users with higher proportions of shared characteristics than in other subgroups.

Many characteristics may affect the formation of subgroups in the MHISG, with some being more relevant than others. The most commonly observed factors influencing the people in interaction are age, gender, and location [3]. These factors are also influential across large-scale online social networks [4]. Specifically, in the domain of MHISGs, there are other factors, which may be important. Different mental health conditions are characterized by different symptoms and experiences [8]. From a psychological perspective, these are fundamental distinctions and they form the basis for different treatments. One might hypothesize that users in the MHISG with similar health concerns would seek to interact with each other, that is, people with depression concerns would provide peer-support to other people with depression, and not anxiety. However, people engaging in peer-support through MHISGs have the autonomy to interact with whomever they choose. These naturally occurring dynamics are currently unknown and a greater understanding of this area is needed. This information may empower community managers to take informed decisions concerning the design of MHISGs. Understanding these natural inclinations also provides a basis for future research to design studies and form hypotheses about relevant factors, which if altered, may affect the outcome of participation and subsequently the potential utility of these communities.

To determine user grouping among the social network of the MHISG, it is recommended to conduct an analysis of its modularity [9]. Modularity is a measure that identifies subgroups in a social network by applying an algorithm designed to find a structure, which optimizes the number of communications within each module compared with the number of communications between different modules. The result of such an algorithm is the assignment of nodes (users) to modules (subgroups), which have a greater density of edges (communications) between them compared with nodes in other modules. It may be possible to use this algorithm in order to determine subgroups of users who engage in higher amounts of peer-support with each other than other users. Using these groups as an outcome, it may be possible to determine whether certain user characteristics are associated with those groupings. To the best of our knowledge, no study has yet investigated modularity in the MHISG. This study aims to determine the community structure of the MHISG through modularity and to explore the user characteristics associated with the resulting structure.

## Methods

### BlueBoard

The data used in this study were obtained from the publicly available Internet support group—BlueBoard (blueboard.anu.edu.au) established by the National Institute of Mental Health Research at the Australian National University. BlueBoard users must register and provide consent for their data to be used for research in order to participate in the MHISG. Peer-to-peer discussion on BlueBoard takes place anonymously via forum postings, which cover a range of topics, including depression (38.8% of posts), bipolar disorder (18.4%), generalized anxiety disorder (5.0%), chitchat and general discussion (22.1%), and other topics (15.7%). Posts dated between October 1, 2008, and May 23, 2014 were included in this study and were in a thread with posts given by two or more users (*n*=130,582 by 2652 users). BlueBoard is moderated by paid personnel who monitor content and enforce rules, for example, by editing posts to remove personally identifying information. BlueBoard moderators do not operate as facilitators of conversation, but post content occasionally regarding rules or other administrative matters. Moderator posts (*n*=352 by 10 moderators) were not included in the analysis. Data collection procedures were approved by The Australian National University Human Research Ethics Committee.

*Measures.* User characteristics included age (measured in 5 year brackets, eg, 25–29); gender (female, male); type of user (consumer, carer, other); location (capital city, other city, rural or remote region); registration date; and the number of posts in each of the subforums of BlueBoard (depression, generalized anxiety, social anxiety, panic disorder, bipolar disorder, obsessive compulsive disorder, borderline personality disorder, eating disorders, carers, general (eg, “chit chat”), and suggestions box).

Age, gender, location, and type of user were self-identified at the time of registration on BlueBoard. The last recorded activity of users was not more than 1 month or 1 year post registration for 86 and 97% of users respectively, thus suggesting that the data likely remained accurate for the majority of users throughout the period of the study. Data on age was grouped into three categories (<25, 25–34, >34) for the analysis to eliminate singularities in the Hessian matrix occurring in brackets above 60 years, with low counts. The three age categories contained approximately one-third of the users each. The term “consumer” refers to a person with depression, anxiety, or other mental health problems, and the term “carer” refers to a nonprofessional carer. The frequency of the posts in each of the subforums was tallied during the entire study duration, that is, from October 1, 2008 to May 23, 2014 for all subforums except the obsessive compulsive, borderline personality, and eating disorder forums, which were established on the June 1, 2009, March 1, 2010, and July 30, 2012, respectively.

### Data Analysis

*Modularity.* Social network analysis was undertaken using Gephi 0.8.2. software [10]. Edges within nodes were defined as undirected communications between each user who had posted in the same thread. The modularity algorithm utilized was the widely-used Louvain method [11], which has the fastest computational time of any comparable algorithm and excellent performance in detecting communities [12]. The resolution was set to the default value 1.0 as this provided the highest modularity score (0.273).

*Multinomial Logistic Regression.* A multinomial logistic regression analysis was conducted to determine the user factors that were significantly associated with the modularity outcome. There were 449 individuals who left at least one of the demographic questions unanswered while registering for BlueBoard. Little’s Missing Completely at Random test was not significant, indicating that the data was missing completely at random; accordingly, they were not included in the analysis.

*Visualization.* In order to explore the results patterns, graphs of the data underlying significant effects were created using pivot tables and charts in Microsoft Excel. To further explore the temporal factor associated with the registration date, a dynamic social network analysis was conducted. This required a timestamp to be associated with the creation of each edge in the social network. The time associated with the creation of each edge was the time a user first posted content in a thread. This edge was created only between the new user and users who had already posted in the thread. A visualization of the edges being created between nodes was generated using Gephi 0.8.2. software and TechSmith Jing screen recording tool [13].

## Results

### Modularity

The modularity algorithm produced 11 separate modules (See Table 1), out of which 6 modules contained less than 10 users. The latter modules were isolated from the giant component of the social network as they involved threads in which only new users not connected to the larger social network posted. The remaining 5 modules comprised between 328 and 954 users, which made 1977 and 67,590 posts, cumulatively. All subsequent analyses are concentrated on these five main modules as outcomes.

**Table 1:** Module sizes

Module	*N* (%)	Posts
1	434 (16.4)	1977
2	954 (36.0)	15,954
3	393 (14.8)	39,720
4	525 (19.8)	67,590
5	328 (12.4)	5300
6	8 (0.3)	27
7	2 (0.1)	2
8	2 (0.1)	2
9	2 (0.1)	5
10	2(0.1)	3
11	2 (0.1)	2
Total	2652 (100)	130,582

### Multinomial Logistic Regression

Multinomial logistic regression is used to finds the odds of being allocated to each of the different dependent variable outcomes based on a number of factors as predictors. In this analysis, the outcomes were the five different modules. One of the outcomes should be used as a reference category for comparison with the other outcomes. In this case, we chose to use Module 4 because its users had contributed the highest number of posts. This decision was made before obtaining any knowledge regarding the number order, we labeled them with. In multinomial logistic regression each of the factors are used to predict the relative odds of persons from the reference group and the comparison group being allocated to each of the two groups as the predictive factors change. In this analysis, several significant effects were found and each of the parameter estimates is shown in Table 2. In this table, the odds ratios, which are the exponents of B, show the relative odds of being allocated to each outcome group as compared with the reference module (thus Module 4 is not included).

With respect to all independent variables in the analysis as predictors of the modularity outcomes, for each unit change, the odds of a person being allocated to the comparison group as opposed to the reference group changes by a factor of the odds ratio. As such, an odds ratio of < 1 indicates that as the score of the predictor increases, the odds of a person being included in the comparison module decreases. An odds ratio > 1 indicates that as the score of the predictor increases, the odds of a person being included in the comparison module increases.

Overall, the final model fits the data significantly better than the null model (Chi square = 4146.4, *p*<.001). The classification accuracy of the model was 84.2% and the effect size was large (Nagelkerke *R* ^2^= 0.891). The addition of one variable, registration date, improved the model classification accuracy by 46.3%. Without this variable, the effect size was much smaller (Nagelkerke *R* ^2^= 0.119).

**Table 2:** Significant parameter estimates for the multinomial logistic regression of registration date, age, gender, location, type of user, and frequency of posts in the subforums on the dependent variable modularity.

Module^a^	Predictor	B	Standard error	Wald	*p* ^b^	Odds ratio
1	Registration date	-0.033	0.001	620.975	<.001	0.968
	Subforum: depression	-0.048	0.018	7.063	.008	0.953
	Subforum: carers	-0.782	0.280	7.834	.005	0.457
2	Registration date	-0.022	0.001	403.895	<.001	0.978
	Subforum: generalized anxiety disorder	-0.082	0.028	8.402	.004	0.921
	Subforum: borderline personality disorder	0.069	0.026	7.272	.007	1.071
	Subforum: suggestions	0.437	0.217	4.061	.044	1.548
3	Registration date	-0.009	0.001	171.921	<.001	0.991
	Subforum: suggestions	-0.165	0.075	4.750	.029	0.848
5	Registration date	0.008	0.001	149.971	<.001	1.008

^a^Module 4 was used as the reference category

^b^All effects degrees of freedom = 1

*Registration Date.* There was a significant parameter estimate for the relationship between registration date and each of the module outcomes as shown in Table 2. For comparing Modules 1–3 with the reference group, the odds ratios of registration date have values < 1. This indicates that a person would be 0.968, 0.978, and 0.991 times as likely to be included in the groups 1–3, respectively, compared with the reference group (Module 4) for each day post registration. The opposite was true for Module 5 relative to the reference group. This indicates that a person would be 1.008 times more likely to be included in Module 5 than Module 4 for each day post registration.

*User Characteristics.* Across the three demographic variables and user type, there were no significant parameter estimates.

*Frequency of Posting in Subforums.* Based on the frequency of posts in the 11 different subforums, there were 6 significant parameter estimates across 5 different subforums. These are shown in Table 2. For the comparison of Module 1 with the reference group, the odds ratios reveal that posting more in either the depression subforum or the carers subforum indicated that a person was more likely to be included in the reference group. For the comparison of Module 2 with the reference group, the odds ratios reveals that posting more in the generalized anxiety disorder subforum indicated that a person was more likely to be included in reference group. The opposite was true for posting in the borderline personality disorder subforum and suggestions subforum. For the comparison of Module 3 with the reference group, posting more in the suggestions subforum indicated that a person was more likely to be included in the reference group. There were no significant parameter estimates for the frequency of posts in subforums in Module 5.

### Visualization

**Figure 1:**
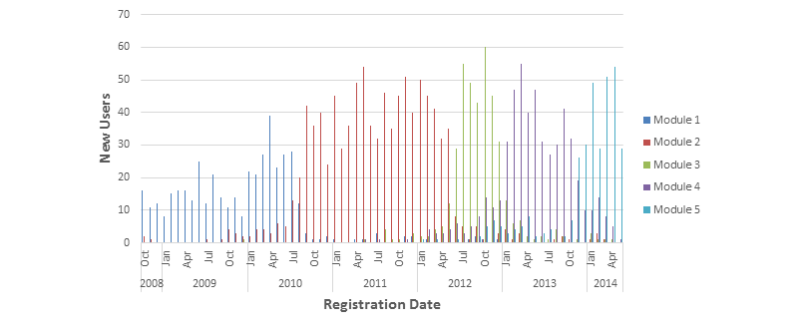
Number of new users who registered each month between October 2008 and May 2014, grouped by module.

*Registration Date.* The graph shown in Figure 1 displays the number of users who registered with BlueBoard during each month from October 2008 to May 2014. Users are grouped by module. This graph supports the pattern of results found in the regression analysis. It shows that the five modules have five sequential time periods in which most of the users who signed up during that period were classified within that group. The distribution of new registrations in each of the four most recent subgroups loosely resembles a normal distribution.

**Figure 2:**
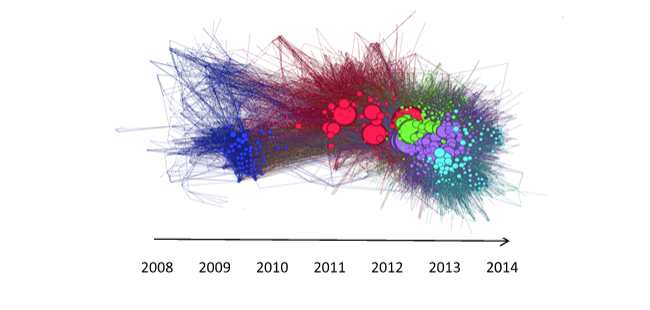
Graphical representation of the sequence of edges created between nodes. Each node is represented by a colored circle, and the nodes are colored according to their module. The size of each node corresponds to its degree (number of connections with other nodes). The layout was determined by the algorithm ForceAtlas 2 [15]. This algorithm places nodes, which have more edges between them, closer together. The arrow on the graph gives a general indication as to the order in which new edges are added to the network as time progresses.

A video showing the sequence in which edges were created between nodes is available as Multimedia Appendix 1: BlueBoard social network growth time lapse. A graph representing this dynamic visualization is displayed in Figure 2. Both show the progression of new communications occurring between users of BlueBoard, primarily between users of the same subgroup during each era progressing from 1 to 5.

### Further Investigation of Registration Date

Since modularity was so strongly associated with registration date, we initiated further analyses to investigate the other factors associated with registration date that might explain the modularity pattern. Based on research, which suggests that online community development follows a life-cycle [14] and that certain “core users” play a vital role from the inception of that development [15,16], we hypothesized that there may be highly active and central users whose registration date is earlier than the majority of other users in each module. For this, we tested whether the top 10 users in each module, ranked by (1) total post frequency and (2) eigenvector centrality (a measure of network centrality, which identifies the most influential nodes [17]), registered significantly earlier than the median registration date for each module. The results of these analyses are presented in Table 3. For total frequency of posts, we found that on average the top ranked users registered significantly earlier than the median registration time in all five modules (*α* < .05). The case for eigenvector centrality was similar, except for the first module. This occurred despite the fact that, across BlueBoard as a whole, there was no significant difference between the average registration date of the top 10 users and the median for either total post frequency (*p*=.40) or eigenvector centrality (*p*=.39). In addition, there was no correlation between total post frequency and registration date (Spearman rho = 0.01, *p*=.60). Contrary to the pattern in each module, there was a significant positive correlation between registration date and eigenvector centrality (Spearman rho = 0.37, *p*<.001).

**Table 3:** T-tests of the mean difference (days) between the median registration date in each module and the average registration date of the top 10 users ranked by (1) total post frequency and (2) eigenvector centrality.

	Total post frequency			Eigenvector centrality		
Module	Mean difference (days)	*t*	*p* ^a^	Mean difference (days)	*T*	*p* ^a^
1	272	-3.56	.006	140	-1.15	.28
2	317	-3.34	.009	293	-2.74	.023
3	145	-3.32	.009	123	-2.64	.027
4	347	-5.96	<.001	377	-8.56	<.001
5	55	-2.84	.019	137	-2.85	.019

^a^All effects degrees of freedom = 9

## Discussion

This study constitutes the first social network analysis of a mental health Internet support group in which the community structure was determined quantitatively through analysis of modularity. We investigated whether several user characteristics were associated with the resulting modularity outcome. The findings of this analysis provide a new perspective on how users engage in peer-support in MHISGs.

### Principal Findings

We found that the community structure of the Internet support group BlueBoard comprised five main modules. Although there were several statistically significant parameter estimates across the different factors for this outcome, registration date contributed the most to the predictive power of the model. Statistically and visually, this factor stood out in the results. The pattern of results suggests that BlueBoard has progressed through a series of generations or eras. There were some minor differences in these generations in the degree to which their users posted in different subforums, but these frequencies did not differ substantially from the overall frequencies for BlueBoard reported elsewhere [Personal communication by Kathleen M Griffiths, 2016].

These results shed light on the nature of peer-support in MHISGs. They suggest that people who join the MHISG may communicate most with those who register around the same time. While this is not surprising, an important finding is the fact that registration date takes precedence over other factors such as demographic characteristics and type of mental health issue in predicting group membership in the MHISG. It raises the possibility that the social interactions of MHISGs are not largely affected by these characteristics. However, it is too early to draw a definitive conclusion as other factors may underpin the observed results.

In order to interpret the findings of this study, we considered whether artificial factors may have impelled the observed progression through each of the five subgroups. We considered two salient factors—external advertising and internal structural changes. Advertising of BlueBoard has occurred mainly via links from online mental health information hubs such as MindHealthConnect.org.au and bluepages.anu.edu.au. Following BlueBoard’s establishment, postcard flyers were soon mailed to general practitioners to be displayed in waiting rooms. Subsequently, there has been a gradual increase in the number of user registrations on BlueBoard. Therefore, recruitment did not appear to be a probable explanation. Further, with respect to internal sources, there were three subforums (obsessive compulsive, borderline personality, and eating disorders) that were introduced at different stages after BlueBoard’s establishment. As there has been little uptake of these forums and they do not correlate with the progressions between the five subgroups, we did not consider this to be a probable explanation. We are not aware of any other developments or improvements that may have resulted in the observed findings. For this reason, we focused on the pattern of results involving the date of registration by highly engaged and central users in each module relative to the majority of other users. This pattern suggests that these users may have some role in the formation of this generation-like structure. However, further research is needed to test this hypothesis and to investigate if these findings generalize to other MHISGs.

### Related Research

This study involved the first analysis of its kind for the MHISG. However, we are aware of a study involving an Internet support group for diabetes, which conducted a similar analysis [18]. This study sought to determine if a modularity analysis could be applied to an online health community and generate meaningful results by creating a formula, which was designed to measure the quality of the modularity outcome. This formula was based on the principle of homophily [7], such that greater similarity among the characteristics (eg, diagnosis) of users in each module resulted in a higher score. The study found that the modularity outcome was associated with the number of years since a user was diagnosed with the condition, indicating the time elapse since diagnosis was similar for users within each module. If as might be expected, the time a person takes to join an Internet support group after being diagnosed is relatively invariant; the findings of this study may have implications for our own. We did not measure time since diagnosis in our study. However, it is possible that the significant effect of registration date is confounded with and attributable to time since the diagnosis period. Alternatively, the diabetes study results can be explained by time of registration.

Our results suggested that type of health concern was not strongly linked to modularity outcome. By contrast, Chomutare et al.’s [18] formula produced a higher score for diagnosis of diabetes type rather than the time since diagnosis, indicating that the former is the stronger determinant of the modularity outcome. This apparent difference in results might imply that the nature of peer-support in a mental health group is less strongly determined by specific health concerns than in a diabetes Internet support group. Alternatively, it could indicate that time since diagnosis has a much smaller effect on the modularity outcome as compared with the registration date in a diabetes Internet support group, or both.

### The Role of Highly Active and Central Users in MHISGs

The observed pattern of highly active and central members registering early in each group in our study is consistent with research which suggests that these users play a vital role in the development of the community at an early stage [16]. The broader literature on online health communities report that “core users” engage in activities of building community by, for example, welcoming newcomers and communicating with many different people [16]. This finding was based on action research on the community #hcsmca and was followed by a quantitative study of the same community, which suggested that core users could be identified as those who have the highest frequency of posts and network centrality [15]. A prospective study of a depression Internet support group suggests that these core users are veterans of the community who increasingly become “active help providers” after an initial period in which they are supported by the others in the community [19]. Thus, the findings from the current study interpret that each module represents an era in which several highly active users communicated with many other new users who registered at the same time regardless of whether they had similar characteristics (as measured in this study) or not, and that these core users played a key role in sustaining the community over time.

### Limitations and Future Research

Although BlueBoard contains a range of subforums for different mental health topics, BlueBoard is predominantly used for discussion on depression. Thus, the generalizability of the current findings to other MHISGs is uncertain and in particular the modularity outcome may differ in MHISGs, which have an evenly spread distribution of posts across different mental health conditions. BlueBoard does not contain subforums for all types of mental health issues. Given the possibility that some forums are, therefore, not used for their intended purpose, the pattern of results may differ in MHISGs with a different variety of subforums. A more refined representation of the social network could also be achieved through collection of systematic data on directed communications between users.

The demographic characteristics of BlueBoard users [Personal communication by Kathleen M Griffiths, 2016] are similar to those of depression Internet support groups reported elsewhere [19]. However, the applicability of the current findings to MHISGs comprising members with markedly different demographic characteristics, such as those dedicated to young people, is unknown. MHISGs including medical professionals as moderators and or active participants might also be characterized by markedly different social dynamics. Accordingly, further research focusing on a range of MHISG types is required to gain a greater understanding of the generalizability of the current findings. Future studies may benefit by modularity in MHISGs to collect and analyze a greater array of user characteristics including diagnosis, time since diagnosis, symptom severity, digital skills, and other characteristics that may reveal motivations of the “core users.”

The role of highly active and influential members is an important area for future research. There are multiple ways of measuring participation in an online health community including some specifying peer-leader roles [20]. We used broad measures in this study (posting frequency and eigenvector centrality), which may not capture the specific nature of different individuals’ contribution to the observed results. Future research with a more specific focus may consider other predefined peer-leadership roles such as “hubs” and “community builders,” who being high frequency posters, are also known for connecting many users and maintaining conversation, respectively [20]. In MHISGs where the identity of users is not anonymous, the role of users who act as hubs or bridges across multiple social networks should also be considered [21,22]. Concurrently, it is also important to understand which characteristics are associated with users who take up these roles. We recently conducted a study of BlueBoard to investigate the user characteristics associated with higher engagement than a single post [Personal communication by Kathleen M Griffiths, 2016], with consumers being found to be more highly engaged than carers. Further research is required to investigate the factors predicting the very highest levels of user engagement and other measures of peer-leadership in online health communities [20]. One previous study has compared the characteristics of the top 1% of users (“superusers”) ranked by posting frequency across two smoking cessation Internet support groups and found no differences between them [23]. A study with higher statistical power may be required to detect significant differences and common characteristics among such a small group of users. As super users are communicating with people who have a range of different mental health concerns, it is possible that super users have multiple or more complex diagnoses, which enable them to relate to and support the other bulk of users who have more specific issues or one-time needs for peer-support. Alternatively, they may have conditions such as bipolar disorder, which result in high activity levels with greater engagement in the community.

## Conclusion

The community structure of the Internet support group BlueBoard comprised five main subgroups that occurred in sequence resembling generations of the MHISG. These groups were largely invariant in their demographic characteristics and the extent to which they communicated about different mental health issues. The community structure formation may be related to the contributions of the most active and central users who registered early as compared with other users in each group.

## Appendix

Multimedia Appendix 1 BlueBoard social network growth timelapse.
